# Acoustic Emission Signal Characterisation of Failure Mechanisms in CFRP Composites Using Dual-Sensor Approach and Spectral Clustering Technique

**DOI:** 10.3390/polym15010047

**Published:** 2022-12-22

**Authors:** Michal Šofer, Pavel Šofer, Marek Pagáč, Anastasia Volodarskaja, Marek Babiuch, Filip Gruň

**Affiliations:** 1 Department of Applied Mechanics, Faculty of Mechanical Engineering, VSB—Technical University of Ostrava, 17. listopadu 2172/15, 708 00 Ostrava, Czech Republic; 2 Department of Control Systems and Instrumentation, Faculty of Mechanical Engineering, VSB—Technical University of Ostrava, 17. listopadu 2172/15, 708 00 Ostrava, Czech Republic; 3 Department of Machining, Assembly and Engineering Technology, Faculty of Mechanical Engineering, VSB—Technical University of Ostrava, 17. listopadu 2172/15, 708 00 Ostrava, Czech Republic; 4 Department of RMSTC, Faculty of Materials Science and Technology, VSB—Technical University of Ostrava, 17. listopadu 2172/15, 708 00 Ostrava, Czech Republic; 5 BREBECK Composite s.r.o., Volenská 1718, 739 34 Šenov u Ostravy, Czech Republic

**Keywords:** acoustic emission, CFRP, failure mechanism, spectral clustering

## Abstract

The characterisation of failure mechanisms in carbon fibre-reinforced polymer (CFRP) materials using the acoustic emission (AE) technique has been the topic of a number of publications. However, it is often challenging to obtain comprehensive and reliable information about individual failure mechanisms. This situation was the impetus for elaborating a comprehensive overview that covers all failure mechanisms within the framework of CFRP materials. Thus, we performed tensile and compact tension tests on specimens with various stacking sequences to induce specific failure modes and mechanisms. The AE activity was monitored using two different wideband AE sensors and further analysed using a hybrid AE hit detection process. The datasets received from both sensors were separately subjected to clustering analysis using the spectral clustering technique, which incorporated an unsupervised k-means clustering algorithm. The failure mechanism analysis also included a proposed filtering process based on the power distribution across the considered frequency range, with which it was possible to distinguish between the fibre pull-out and fibre breakage mechanisms. This functionality was particularly useful in cases where it was evident that the above-mentioned damage mechanisms exhibited very similar parametric characteristics. The results of the clustering analysis were compared to those of the scanning electron microscopy analysis, which confirmed the conclusions of the AE data analysis.

## 1. Introduction

Composite materials in many forms have become increasingly popular in recent decades, especially in the medical, automotive, and aerospace industries. In particular, carbon-fibre-reinforced polymer composites have been used in a wide range of sporting activities such as cycling (bicycle frames and accessories), yachting (hull, mast, and other construction elements), or motorsports (bonnet, bike frame, and wheel rims). The main advantages of CFRP composites are that they have a very high strength/weight ratio and that their properties can be customised for a specific purpose by changing the stacking sequence of the individual plies [1]. However, their main drawback is their inability to fail in a ductile manner [2]. Moreover, the final fracture occurs as a result of gradual accumulating damage, which is barely visible on the outside surface [3]. This is one of the main reasons for adopting a suitable method for the comprehensive analysis and characterisation of failure mechanisms in CFRP composites.

Currently, numerous non-destructive testing (NDT) methods, such as ultrasonic testing [4], eddy current testing [5], and X-ray tomography [6], have been applied to detect and/or localise defects in composites. However, these methods have several limitations regarding real-time evaluation, difficult feasibility or the voluminal portion of the material, which can be tested in real time. Hence, the acoustic emission method [7] has been used in a large number of studies [8,9,10,11] because it offers a practical and effective solution compared to the methods listed above. The AE method belongs to a group of NDT methods that are fully supported by international standards for applications in technical practice. The method is highly effective for testing metallic pressure equipment [12], such as pipelines, boilers, seamless steel vessels, autoclaves, and composite vessels, where the modal AE technique is often used [13]. The AE method is based on the detection of acoustic stress waves originating from a material as a result of sudden stress redistribution owing to the present microstructural damage, which is triggered by the applied load [12]. The captured AE signals are then analysed, and they may exhibit different degrees of complexity. These different degrees of complexity are particularly important in the case of composite materials, which have several types of failure mechanisms [14], such as fibre breakage, fibre/matrix debonding, delamination, or matrix cracking.

Numerous studies have been devoted to the characterisation and methodology of failure mechanisms. The most basic approach is parameter-based analysis [15] of the detected AE signals using features such as amplitude, rise time, energy, duration, and frequency parameters in the form of frequency centroids or peak frequencies [16]. This approach is less effective for composite structures, mostly because of the overall complexity of the failure mechanisms, which may occur simultaneously. As a result, the detected AE signals may be associated with more than one failure mechanism [2,17]. One of the major drawbacks of parameter-based analysis is the considerable parameter sensitivity of the geometry, sensor type, or distance between the sensor and the AE source (the influence of signal attenuation) [2].

A more recent approach is signal-based analysis [16], whose development and subsequent application have been highly conditioned by AE instrumentation. In this approach, individually detected AE waveforms are recorded and subjected to frequency analysis, mostly in the form of the Fourier transform [3], wavelet transform [18,19], wavelet packet transform [20], or Hilbert–Huang transform [21].

In the last two decades, supervised/unsupervised clustering and pattern recognition algorithms [22] based on multi-parameter analysis [2] have been adopted in a large number of studies because of their ability to utilise features from both parameter-based and signal-based analyses. In the supervised approach, the input signals are associated with a given class or cluster, which, for example, represents a particular failure mechanism using the training dataset. In the unsupervised approach, signals with similar characteristics are grouped without using the training dataset. The most commonly used clustering method among the unsupervised approaches is k-means [23,24,25], followed by its variants such as k-means++ clustering [26], fuzzy c-means clustering [27,28], or genetic k-means clustering [29], which may be combined with other approaches such as self-organising maps (SOMs) [30] or principal component analysis (PCA) to improve their effectiveness [31].

Although there is a relatively extensive knowledge base of various methodologies for identifying failure mechanisms in CFRP composites, certain issues still arise regarding their overall characterisation. For example, if the failure mechanisms are classified based on the peak frequency, some inconsistent results are obtained, although the same type of AE sensor (WD sensor; Physical Acoustics Corporation) is used [32] (see Table 1). This can be attributed to a number of factors, such as the tested material, specimen geometry, variations in the sensor response, or processing methodology. The greatest variations in terms of peak frequency occur in the case of fibre breakage or fibre pull-out, followed by delamination and fibre/matrix debonding.

The aim of this study is to investigate the characteristics of the failure mechanisms of CFRP composite materials. Two test series are conducted in this study: the first test series involves only one failure mechanism, which is fully characterised, and the subsequent test series involves several types of failure mechanisms. Two wideband sensors [22] are used to monitor the AE activity. Unlike multiresonant/resonant sensors, wideband sensors provide a more accurate characterisation of AE signals in terms of their parameters. The collected AE signals are then analysed using the spectral clustering technique, which utilises the k-means unsupervised pattern recognition approach. Subsequently, the clustering results are associated with the individual types of tests performed, followed by a discussion related to the failure mechanism characterisation, which is additionally supported by scanning electron microscopy (SEM) results.

## 2. Experiments

**Figure 1:**
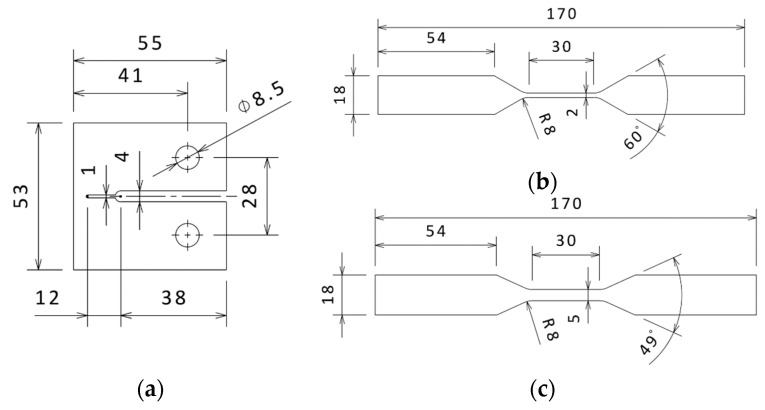
Geometry of the tested specimens [mm]: (**a**) BRCT, CPCT, and UCT samples; (**b**) CPT sample; (**c**) CPTS sample.

The experiments involved two tests: tensile and compact tension (CT) tests. The tensile test was conducted on carbon fibre bundle (CFB) (Havel Composites CZ Company Ltd., Czech Republic) and cross-ply specimens, whereas the CT test was conducted on bulk resin (BRCT), unidirectional (UCT), and cross-ply (CPCT) specimens, which are shown in Figure 1. Note that each sample type was represented by four pieces. The BRCT specimens were made of LH 385 epoxy resin with curing agent H512 (Havel Composites CZ Company Ltd., Czech Republic). The UCT and CPCT specimens were made of high-performance carbon/epoxy prepreg CM-Preg T-C-230/600 CP004 39 with a nominal ply thickness of 0.25 mm. The BRCT and UCT/CPCT plates were cured based on the schedule provided by the manufacturer. Detailed information about the individual specimens is presented in Table 2 and Table 3.

Both tests were conducted on the Testometric M500-50CT universal testing machine in deformation-controlled mode with an upper grip speed of 1 mm/min for the tensile test of the CFB specimens and 0.5 mm/min for the other tests. Additionally, a load cell of 100 N was utilised for the tensile test of the CFB specimens and the CT test of the UCT specimens, whereas a 50 kN load cell was utilised for the CT test of the BRCT and CPCT specimens, as well as the tensile test of the CPT and CPTS specimens.

### 2.1. Acoustic Emission Monitoring

**Figure 2:**
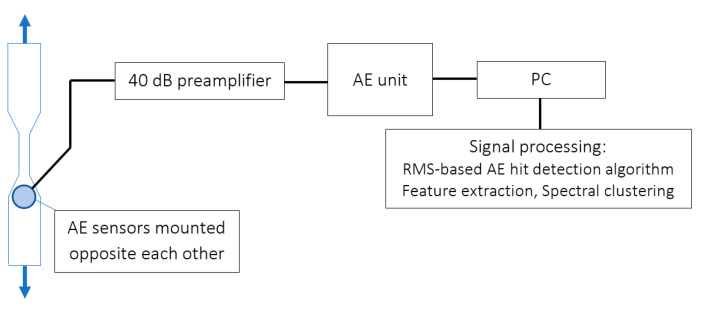
Schematic diagram of the experimental setup.

The AE activity was recorded using two wideband AE sensors, DWC 454 and Fujicera 1045S, which were directly connected to a Vallen AEP5H 40 dB preamplifier. The already amplified signal is then transferred to the AE unit, where basic signal processing takes place, followed by a transfer of the AE data to the PC for the entire data processing procedure (see Figure 2). It must be emphasised that the maximum distance between the axis of the sensor and the area in which the AE activity occurred was not greater than 25 mm. Thanks to this, it was possible to largely neglect the dependence of the frequency spectrum on the distance from the AE source. Both utilised AE sensors are designed as wideband sensors with a usable range from 50 to 1200 kHz, while in the case of Fujicera, it is possible to count on an even wider band due to increased sensitivity at frequencies above 1 MHz. 

**Figure 3:**
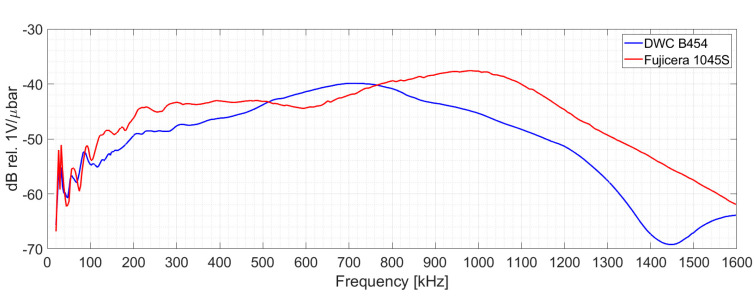
Face−to−face calibration results of DWC B454 and Fujicera 1045S AE sensors using the Vallen Sensor Tester^®^ utility.

Figure 3 displays the results of face-to-face relative calibration of both sensors using the Vallen Sensor Tester^®^ utility (Manufacturer: Vallen Systeme GmbH, Germany). The captured signals were then processed by a Vallen AMSY-6 AE system unit comprising an ASIP-2A signal processor card with sampling frequency and frequency filter set to 10 MHz and 50–1100 kHz, respectively.

Most commercially available AE devices use the fixed-threshold principle for hit detection. The main disadvantage of this principle is the tendency to artificially combine hits that emerge in rapid succession. The reason for this behaviour is that the signal under the given circumstances does not fall below the threshold; therefore, the hit is not interrupted. As a result, such hits contain a number of otherwise separated hits, leading to significant inaccuracies in the characterisation of AE signals in some cases (that is, specimen violation during the tensile test). Although this behaviour can be reduced to some extent by using suitable parameters for the hit separation process or by increasing the sensing threshold, these steps do not lead to the complete elimination of this phenomenon.

In 2017, Shateri et al. proposed a root mean square (RMS) AE hit detection algorithm [15] that can overcome the problems associated with the threshold-based technique by using the information stored in the RMS envelope of the AE signal. Subsequently, the joint-valley peak-finding algorithm is then applied to detect separate events. The functionality of the entire algorithm is not described in this paper, as it has been explained in detail by Shateri et al. [15]. However, we modified this algorithm to further improve its performance.

The RMS-based AE hit detection algorithm uses three functionalities to ensure the detection of possible AE hits, namely two thresholds: one for the RMS and the other for the original AE signal, and the ratio between the present peak and valley within the RMS envelope, which is then used to validate whether the detected AE hit is valid. Shateri et al. [15] derived an expression for calculating a given peak/valley ratio depending on the RMS envelope and the original time signal thresholds. However, we found that this ratio depends on a number of factors, such as the size of the time window for calculating the RMS value, the type of sensor, nature of the AE signal, and the geometry being tested.

Additionally, for a more efficient separation of the AE hits, we implemented a two-level threshold (fixed and floating) for the RMS signal waveform. In practice, this threshold segments potential hits in the first phase using a predetermined fixed RMS threshold, and then, depending on the RMS value of the background noise before the start of the hit, the maximum amplitude of the RMS waveform is set as a new RMS threshold, allowing more efficient hit segmentation.

The procedure above can be applied to wave transients, which are obtained by the continuous recording of waveforms for a given measuring channel or by using a fixed amplitude threshold by the procedure below:

1. The AE activity was recorded at a fixed threshold value of 28 dB_AE_, while the duration discrimination time (DDT) and rearm time (RAT) were set to 100 µs;

Note: According to Vallen documentation, DDT is the period in which no threshold crossing must occur to determine the end of a hit, whereas RAT is the period after which the channel is ready to generate a new hit dataset;

2 The recorded wave transients were subsequently subjected to an additional hit segmentation process with the following parameters: width of the RMS window = 10 µs, fixed value of the RMS threshold = 10 dB_AE_. The floating value of the RMS threshold is given by:
  (1) $$rms_{floating}=rms_{mean}+0.12\left[max\left(rms\right)-rms_{mean}\right]$$
  where rms_mean_ is the mean value of the RMS before the start of the AE hit (mean value from the first 10 samples before the hit start), max(rms) is the maximum value of the RMS recording for the given AE hit, and peak/valley ratio = 0.25 (maximum) for valid pairs exceeding the principal threshold of 28 dB_AE_.

The AE signal parameters [36] or features act as input variables for the subsequent clustering process. These features can be further divided into directly extracted features and those that are calculated during post-processing. Table 4 lists all the features used in this study and their descriptions.

### 2.2. Failure Mechanisms

#### 2.2.1. Tensile Test of Fibre Bundles and Compact Tension Test of Bulk Resin

**Figure 4:**
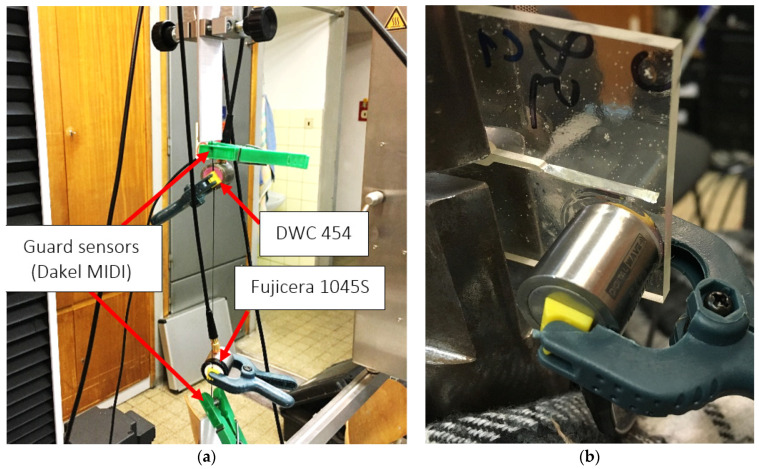
In situ photographs of the (**a**) tensile test of the carbon fibre bundle, (**b**) BRCT specimen, including the present unstable macrocrack.

The AE signals originating from fibre breakage were investigated by conducting tensile tests on the fibre bundles, which were adequately adhered to the clamps using a cyanoacrylate adhesive. To eliminate the possible occurrence of fibre pull-out in the clamps, the peripheral parts of the fibre bundle were equipped with guard sensors (DAKEL MIDI), as shown in Figure 4a. Before the actual evaluation of the AE data from the tensile test results of the carbon fibre bundles, it was necessary to perform data filtering because the failure of carbon fibres, which are not separated by a matrix, has completely different characteristics compared to conventional composites. Moreover, a relatively large percentage of the AE activity comes from the mutual friction of the fibres, which has an increasing tendency, especially when a larger number of fibres fail at the same time. The broken fibres then begin to slide over those that still carry a certain load, with the nature of the detected signal being very similar to that corresponding to the fibre failure. Hence, it was necessary to find a suitable methodology for separating the useful signal (in our case, fibre failure) from the secondary AE. For the given purposes, an evaluation algorithm in which the filtering of AE hits coupled with fibre breakage is based on the detection of a decrease in force over a time course was developed with MATLAB software. The interval of interest is defined by the detection of a negative gradient and the moment of returning the force to its original values, which includes the extension of both borders by the force sampling period (100 ms). Only hits falling within these intervals are subsequently analysed. The only shortcoming of the given approach is that it is not possible to filter out the secondary AE emission if it occurs simultaneously with a fibre break, which is associated with interval reporting force drops.

**Figure 5:**
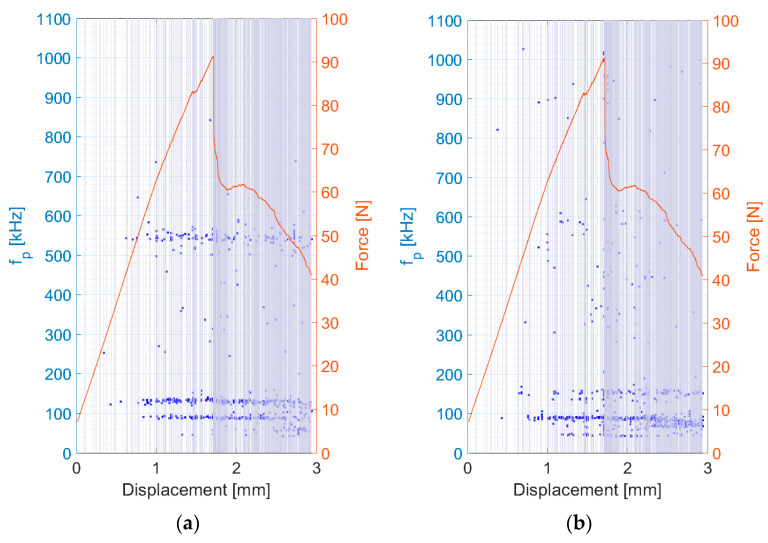
Peak frequency and force versus displacement results of the fibre bundle tensile test. (**a**) DWC 454 AE sensor; (**b**) Fujicera 1045S sensor.

Figure 5 shows the relationship between the peak frequency and force versus displacement for the representative specimen of the carbon fibre bundle after the filtering process of the results from both AE sensors (the blue highlighted segments indicate the detected force dips). The results from the DWC 454 AE sensor showed that, particularly in the initial phase, AE signals with a peak frequency above 500 kHz existed (see Figure 5a). After reaching the maximum force, the failure of the fibres began to appear on a slightly wider peak frequency scale. This was mainly due to the superposition of signals originating from both the fibre breakage and the above-mentioned friction between the fibres in the bundle, which occurs along the entire length of the bundle.

A similar scenario was observed in the results obtained from the Fujicera 1045S AE sensor, where the upper limit of the peak frequency interval shifted to 1000 kHz, whereas the lower interval started at approximately 300 kHz (see Figure 5b). For the subsequent clustering process, we considered only the AE hits from both sensors with a peak frequency higher than 400 kHz, which seemed like a reasonable compromise for both datasets.

Regarding the CT tests of the bulk resin specimens, the only mechanisms observed were matrix microcracking and macrocracking, which have the characteristic of brittle fracture. Because the initiation process of microcracks is practically undetectable, the only mechanism covered by the AE activity is the unstable propagation of macrocracks associated with an overall collapse of the structural integrity (see Figure 4b).

#### 2.2.2. Compact Tension Test of Unidirectional Specimens

**Figure 6:**
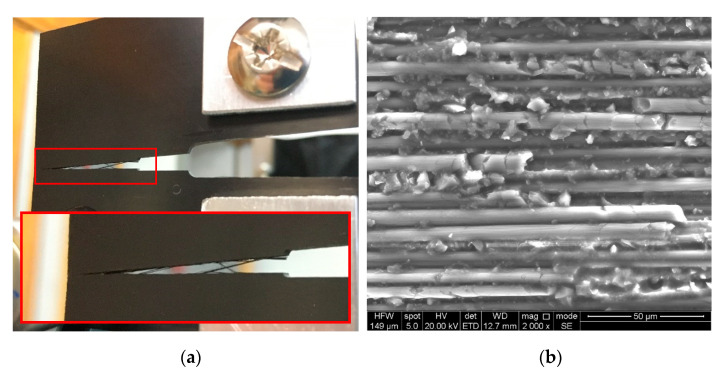
In situ photograph of the CT test of UCT specimens. (**a**) Detail of the fibre-bridging process; (**b**) SEM image of the fracture surface of a UCT representative specimen.

The CT tests of the UCT specimens revealed the presence of several failure mechanisms, namely matrix microcracking, fibre/matrix debonding, and fibre failure, as shown in Figure 6b. After the initial phase of microcrack initiation and coalescence, the propagation of the macrocrack began, which was characterised by a sudden drop in force. Meanwhile, the fibre-bridging phenomenon [39] (Figure 6a) was observed in the CT tests of the UCT specimens, indicating that this phenomenon significantly contributes to fibre/matrix debonding and fibre failure, which agrees with the results of Gutkin et al. [34].

#### 2.2.3. Compact Tension and Tensile Test of 0–90° Cross-Ply Specimens

The 0–90° cross-ply specimens were subjected to tensile and CT tests, and their loading characteristics were compared. The CT test was completed over a longer period, during which the final stage of the structure violation was monitored. Meanwhile, the tensile test was characterised by an abrupt collapse, during which a relatively large amount of information was lost owing to the sudden release of energy and the superimposition of the individual AE signals.

**Figure 7:**
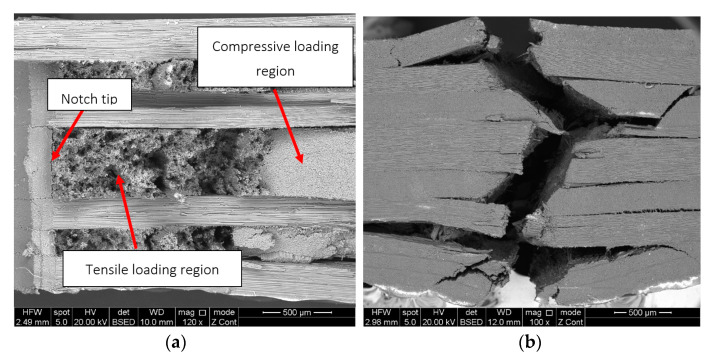

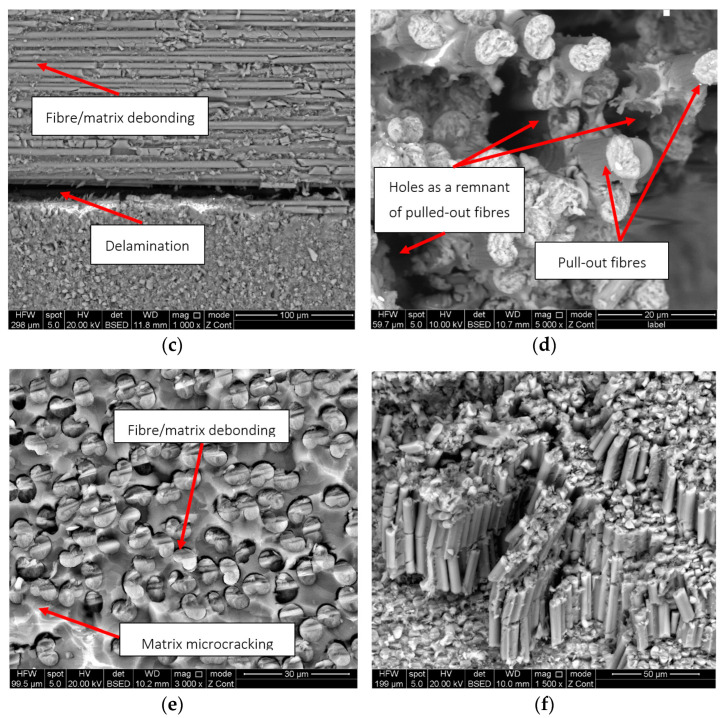
SEM images of a CPCT representative specimen: (**a**) general view, (**b**) structure collapse due to compressive load, (**c**) fibre pull-out, (**d**) delamination and fibre/matrix debonding, (**e**) fibre/matrix debonding and matrix microcracking, (**f**) fibre failure.

The SEM analysis of representative samples from both types of tests revealed the occurrence of delamination, fibre/matrix debonding, matrix cracking, fibre failure, and fibre pull-out. The CPCT samples showed the presence of regions with tensile and compressive loading characteristics (Figure 7a). Compressive loading was mainly responsible for the delamination process at the back part of the representative sample (Figure 7b,c), including fibre/matrix debonding and matrix microcracking (Figure 7e), followed by fibre failure (Figure 7f). In contrast, the region with tensile loading characteristics had noticeable traces of fibre pull-out and fibre breakage (Figure 7d), followed by regions showing fibre/matrix debonding and delamination. Similar observations were found for the tensile test of the CPT samples, and the same characteristics were observed for the above-mentioned failure mechanisms.

#### 2.2.4. Tensile Test of ± 45° Cross-ply Specimens

**Figure 8:**
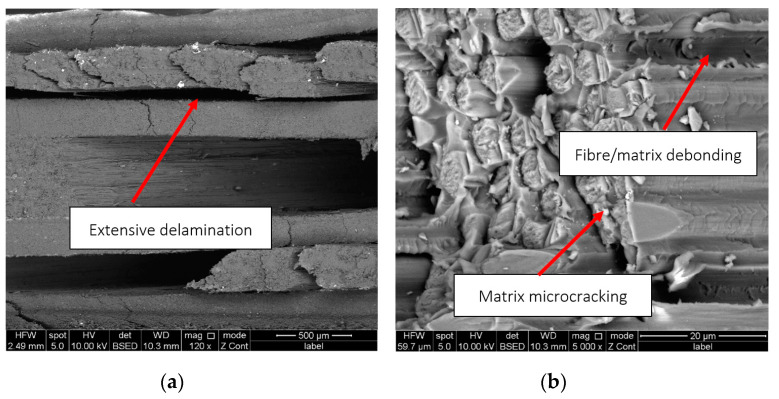
SEM images of a CPTS representative specimen: (**a**) delaminated plies, (**b**) matrix microcracking and fibre/matrix debonding.

The SEM analysis of the CPTS specimens revealed the presence of delamination (Figure 8a), matrix microcracking (Figure 8b), and fibre/matrix debonding. 

In addition to these failure mechanisms, which occurred to a large extent, fibre failure was also observed, but not to the same extent as in the case of the aforementioned mechanisms.

## 3. Pattern Recognition Technique

The amount and type of the selected AE signal features, which are further processed using a supervised/unsupervised pattern recognition approach, significantly influence the final results in terms of assigning the respective AE hits to individual clusters. In the literature, it is possible to find approaches based on the use of only basic signal features [34], such as amplitude, risetime, or energy, that can be obtained directly from waveform analysis, and methodologies that incorporate a more branched number of extracted/computed AE signal features [40,41]. Theoretically, for the clustering process, as many AE signal features as possible should be used. However, based on the performed preparatory analyses, it has been proven to be very effective to use more or less fundamental AE signal parameters (see Table 4), from which the suitable ones will be then extracted entering the clustering process.

### 3.1. Feature Selection

The first step is to extract important features from the currently considered features (see Table 4). The extraction process involves the Laplacian score technique, which was introduced in 2005 by He et al. [42]. The Laplacian score is associated with each feature and reflects its locality-preserving power, which means that two data points are most probably related to the same topic if they are close to each other [42]. According to He et al. [42], the local structure of the data space in classification problems is more important than its global counterpart. To maintain this assumption, the Laplacian score algorithm utilises the nearest neighbour graph [42]. The Laplacian score ranges from 0 to 1, and important features should have a Laplacian score greater than 0.9 [41,43,44].

The calculation of the Laplacian scores was conducted using the *fsulaplacian* function of MATLAB (R2021a version). The entire process of calculating the Laplacian score is divided into four steps [42,45], which are briefly presented below:

1. For a given matrix **K**_n × p_ containing normalised input data, construct the nearest neighbour graph. Afterwards, define pairwise distances *d_i,j_* for all points in the neighbourhood;

Note 1: We used the min-max normalisation approach;

Note 2: The rows of the input data matrix **K** correspond to the observations, whereas the columns correspond to the features;

Note 3: The *fsulaplacian* function provides various distance metrics for calculating pairwise distances. In our case, we used the Euclidian distance metric;

2. Generate the similarity matrix **S** using the kernel transformation $S_{i,j}=e^{{\left(\frac{-d_{i,j}}{\sigma }\right)}^{2}}$, where σ is the scale factor for the kernel and $d_{i,j}$ is the pairwise distance between two arbitrary nodes *i* and *j* ($d_{i,j}$ refers to the Euclidian distance in our case);
3. Perform the centring of each feature using its mean ${\tilde{k}}_{r}=k_{r}-\frac{k_{r}^{T}D_{g}I_{v}}{I_{v}^{T}D_{g}I_{v}}I_{v}$, where $D_{g}$ is the degree matrix and $I_{v}={\left[1,\cdots ,1\right]}^{T}$;
4. For each feature, compute the score $s_{r}=\frac{{\tilde{k}}_{r}^{T}S{\tilde{k}}_{r}}{{\tilde{k}}_{r}^{T}D_{g}{\tilde{k}}_{r}}$;

### 3.2. Clustering Technique

Supervised/unsupervised pattern recognition approaches are an integral part of several scientific studies within this field. The most used approach is the k-means clustering technique [15,23,25] and its derivatives, such as k-medoid, fuzzy k-means, genetic k-means, or k-means++ [32,46], which may be combined with other techniques such as SOM, competitive neural networks [34], or PCA [32].

An alternative method within this area is the spectral clustering approach, which has become one of the most modern and popular techniques [47]. Spectral clustering has a simple implementation and outperforms classical approaches such as the k-means algorithm in most cases [47,48]. The technique is a graph-based approach that constructs a graph, finds its Laplacian matrix, and uses the matrix to obtain *h* eigenvectors, which are then used to split the graph in *h* ways [49]. In this study, spectral clustering was performed in MATLAB (R2021a version) using the *spectral cluster* function with the following implementation [48,49]:

1. Assemble the similarity graph for a given set of points defined by *X*;
2. Calculate the similarity (or adjacency) matrix **S**, with $S_{i,j}=e^{{\left(\frac{-d_{i,j}}{\sigma }\right)}^{2}},$ where σ is the scale factor for the kernel and $d_{i,j}$ is the pairwise distance between two arbitrary nodes *i* and *j* ($d_{i,j}$ is the Euclidian distance in our case);
3. Construct the Laplacian matrix L=D−S, where D denotes the degree matrix;
4. Find $g_{1},g_{2}\cdots g_{h}$, the *h* smallest eigenvectors of matrix L, and form matrix $G=\left[g_{1},g_{2}\cdots g_{h}\right]$ by stacking the eigenvectors into columns;
5. Treat each row in G as a point and perform k-means clustering;
6. Assign the original points in *X* to the same clusters as their corresponding rows in G.

Because most clustering techniques require a certain number of user-supplied parameters, a validity metric must be adopted, which will provide the user with information related to the effectiveness of the clustering process. The most well-known cluster validity metrics include the Dunn index, the Davies–Bouldin index, the SD validity index, the S_Dbw index, and the silhouette coefficient [50]. These validity metrics can be further categorised based on the assessment of the degree of intra-cluster cohesion, degree of inter-cluster separation, or both, referred to as a hybrid approach, which comprises the Davies–Bouldin index and the silhouette coefficient [50]. In our case, we chose the silhouette coefficient as a cluster validity metric partly due to the nature of the acquired datasets and because we require the evaluation of both the cohesion of the objects within clusters and the separation between clusters. Moreover, the normalisation property of the silhouette coefficient enables the comparison of the results from different datasets (datasets from DWC 454 and Fujicera 1045S AE sensors). The silhouette value $s\left(i\right)$ for individual data points $x\left(i\right)$ within a given dataset is defined as follows:

(2) $$s\left(i\right)=\frac{b\left(x_{i}\right)-a\left(x_{i}\right)}{max\left(a\left(x_{i}\right),b\left(x_{i}\right)\right)}$$

where $a\left(x_{i}\right)$ is the average intra-cluster distance of point $x_{i}$ related to all other points within the concerned cluster and $b\left(x_{i}\right)$ is the minimum of the average inter-cluster distances of point $x_{i}$ to all points in each other cluster [50]. The silhouette coefficient can take values from −1 to 1, and the larger the coefficient, the better the matching of the point to its own cluster. The silhouette coefficient SC represents the mean of the silhouette values over all data points [51]:

(3) $$SC=\tilde{s}\left(i\right)$$

## 4. Results and Discussion

The results and their related analysis include two datasets from the Fujicera 1045S and DWC 454 AE sensors. Prior to the cluster analysis, both datasets were subjected to additional filtering with a minimum acceptable hit energy of 10 aJ to avoid irrelevant indications such as random overshoots in the low-frequency range. As mentioned above, for the results of the fibre bundle tensile tests, peak frequency filtering was additionally performed, with the lower limit for hit acceptance being 400 kHz. Furthermore, suitable feature candidates were selected using the Laplacian scores technique, and only those features with a Laplacian score higher than 0.9 were considered important features. Clustering was then performed on a data file, considering the selected features that corresponded to all the measurements made by the given sensor (Fujicera 1045S or DWC 454). Subsequently, the optimal number of clusters was evaluated with the silhouette coefficient. The results from each test and the relevant sensor are presented for a representative sample from each sample series, which included a total of four tests. The following dependencies are provided:

1. Relationship between amplitude and force versus displacement (**a**);
2. Relationship between peak frequency and force versus displacement (**b**);
3. Relationship between AE hit energy versus peak frequency (**c**).

where the displacement variable represents the relative position of the grip with respect to its initial position.

**Figure 9:**
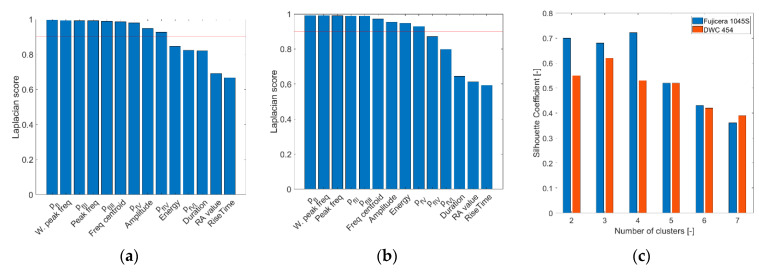
Laplacian score results from the (**a**) Fujicera 1045S and (**b**) DWC 454 sensors; (**c**) silhouette coefficient.

Figure 9 shows the results of the Laplacian score analysis and the silhouette coefficient for both datasets as a function of the number of clusters. Important features in the Fujicera 1045S dataset included nine parameters mainly related to the frequency domain, namely, peak frequency, weighted peak frequency, frequency centroid, partial power in frequency segments *f_I_–f_V_* (see Table 4), and amplitude. The DWC 454 dataset showed very similar results to its Fujicera 1045S counterpart. The group of nine important features also included energy instead of partial power in the frequency interval *f_IV_* (see Figure 9a,b). The optimal number of clusters was three (SC = 0.62) for the DWC 454 AE sensor dataset and four (SC = 0.72) for the Fujicera 1045S AE sensor dataset. In both cases, because the value of the silhouette coefficient was above 0.6, an acceptable level of clustering quality was reached.

**Figure 10:**
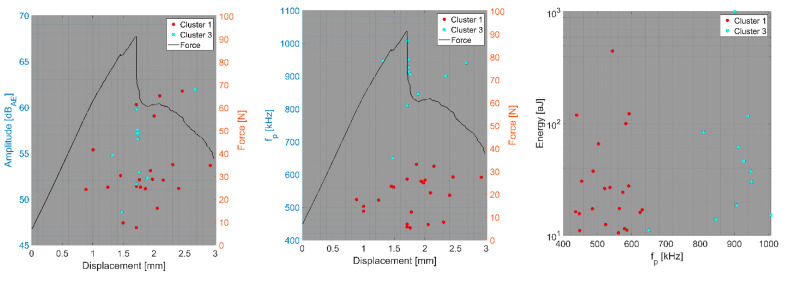
Clustering results for a CFB representative sample using a spectral clustering algorithm (Fujicera 1045S dataset).

**Figure 11:**
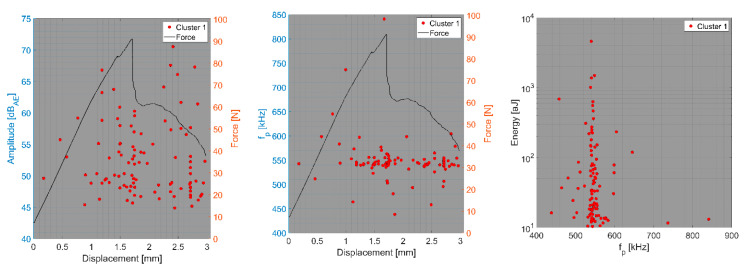
Clustering results for a CFB representative sample using a spectral clustering algorithm (DWC dataset).

**Figure 12:**
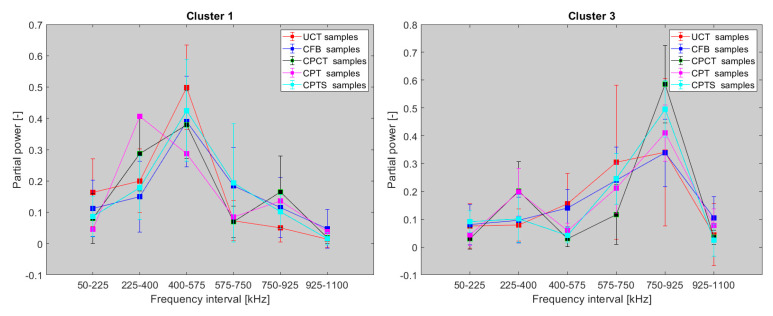
Mean values of the partial power variable corresponding to individual frequency intervals (Fujicera 1045S dataset, clusters one and three).

In the first step, we focus on the tensile test of CFB specimens and the CT test of BRCT specimens. The number of detected AE hits in both tests was relatively small compared to that in the other tests. In the DWC 454 dataset, only one cluster per test type was observed, whereas in the case of the Fujicera 1045S dataset, two clusters were observed for the carbon fibre breakage mechanism, whose peak frequency does not depend on energy or amplitude (see Figure 10a–c). Moreover, the DWC 454 AE sensor revealed carbon fibre breakage at a much narrower frequency interval, that is, 400–600 kHz (see Figure 11), with a somewhat wider range of AE hit energy, whereas the Fujicera 1045S AE sensor exhibited a wider frequency span for a given failure mechanism, that is, 400–1000 kHz. The results of the other tests within the Fujicera 1045S dataset point to the fact that the AE hits belonging to cluster three may also represent fibre pull-out. In order to distinguish the above-mentioned two mechanisms, we have utilised the already calculated partial power variable in individual frequency intervals. Figure 12 shows the mean values of the partial powers, including their standard deviations (represented by error bars), for the tensile/CT tests covered by the Fujicera 1045S dataset. Let us now consider the curve belonging to the CFB samples for both clusters as a reference. Within the cluster one results, which are associated with fibre breakage, share the same character CPTS samples and, to a certain extent, the CPCT samples. The wave transitions belonging to cluster one in the case of the UCT samples tend to be more low-frequency-like, which is particularly true for CPT samples. The interesting aspect of the data affiliated with cluster three is that it provides a certain prescription for the data coupled with fibre breakage. Comparing the UCT sample curve to the CFB sample curve, the AE hits coupled to these two different tests showed the same failure mechanism—fibre breakage. Meanwhile, the CPCT/CPT/CPTS curves exhibited a higher partial power in the 750–925 kHz frequency interval, indicating that these AE hits are associated with different failure mechanisms. 

Therefore, a subsequent proposal is to filter the AE hits within cluster three using the mean value and standard deviation of the partial power in the 750–925 kHz frequency interval corresponding to fibre breakage (CFB samples). The cluster of three AE hits with a higher value of partial power in the given frequency segment belong to the fibre pull-out mechanism.

**Figure 13:**
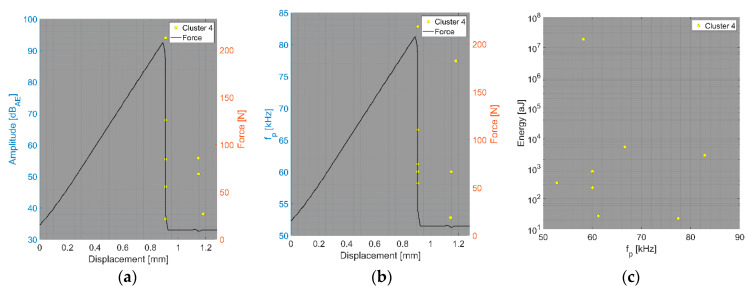
Clustering results for a BRCT representative sample using a spectral clustering algorithm (Fujicera 1045S dataset).

**Figure 14:**
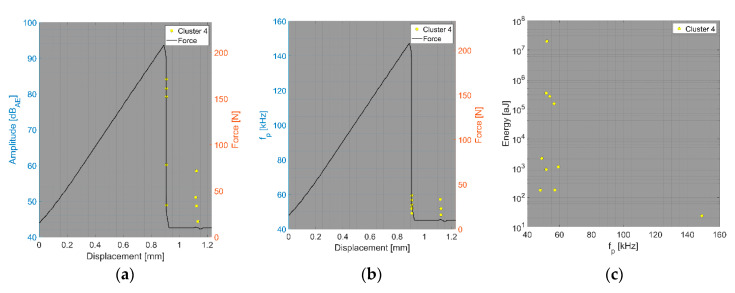
Clustering results for a BRCT representative sample using a spectral clustering algorithm (DWC dataset).

The parametric description of AE hits belonging to matrix cracking (Figure 13 and Figure 14) was almost identical for both datasets with respect to the monitored quantities. In summary, matrix cracking produced AE hits in their macro form with very high amplitudes and high energy rates, with peak frequencies not exceeding 100 kHz.

**Figure 15:**
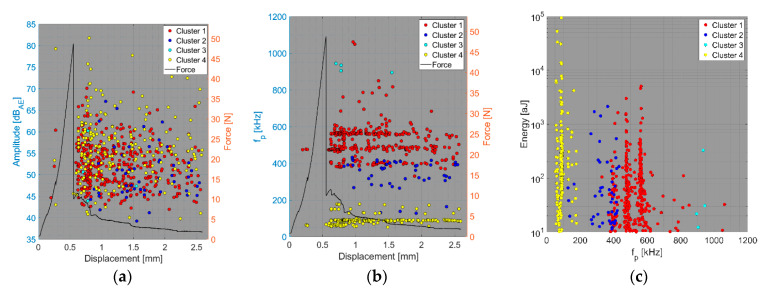
Clustering results for a UCT representative sample using a spectral clustering algorithm and secondary filtering using a partial power variable (Fujicera 1045S dataset).

**Figure 16:**
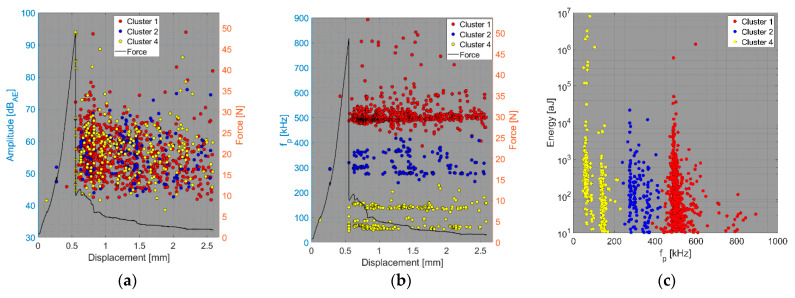
Clustering results for a UCT representative sample using the spectral clustering algorithm (DWC dataset).

**Figure 17:**
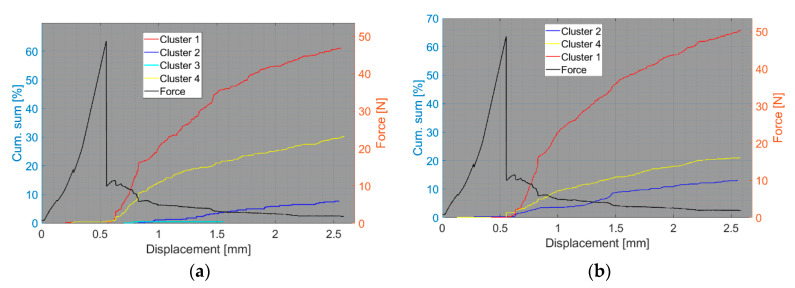
Cumulative sum of hits of individual clusters, including force versus displacement—UCT samples: (**a**) Fujicera 1045S dataset; (**b**) DWC 454 dataset.

We now consider the groups of samples with several failure mechanisms. For the CT test of the UCT specimens, the SEM analysis of the fracture surfaces revealed fibre breakage, fibre/matrix debonding, and matrix macro/microcracking, including the fibre-bridging phenomenon (see Section 2.2.2.). In the clustering results, both datasets exhibited relatively similar characteristics. The AE activity occurred at the moment of macrocrack propagation, which was characterised by a sudden decrease in force. The clustering process resulted in the identification of four clusters in the Fujicera 1045S dataset (Figure 15), whereas the dataset belonging to the DWC 454 AE sensor revealed three clusters (Figure 16). Cluster four represents matrix cracking, which can be further divided into two sub-types: AE hits with peak frequencies lower than 100 kHz, which come from a wider range of energy values, and AE hits with peak frequencies between 100 and 200 kHz, which have lower energy values (see Figure 15b,c and Figure 16b,c). Based on the cumulative AE hits for individual clusters (Figure 17a,b), it is quite evident that the cluster four activity, especially its low-frequency part, occurs immediately after the onset of macrocrack propagation. Another clearly assignable source is fibre failure, represented by cluster one (in both datasets). It has to be noted that cluster one is represented in the greatest abundance—see Figure 17a,b. Cluster two then represents fibre/matrix debonding, which was also confirmed by the SEM analysis for this type of sample (Figure 6b).

Note that the Fujicera 1045S dataset shows a partial intermingling of clusters one and two, i.e., fibre failure and fibre/matrix debonding, which is most likely due to the flatter frequency spectrum compared to the DWC 454 AE sensor in the given range—see Figure 3. 

**Figure 18:**
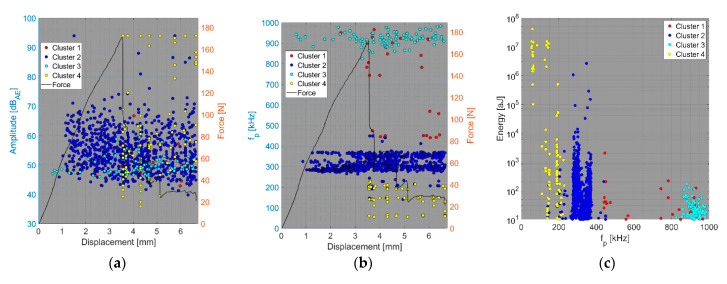
Clustering results for a CPCT representative sample using a spectral clustering algorithm and secondary filtering using a partial power variable (Fujicera 1045S dataset).

**Figure 19:**
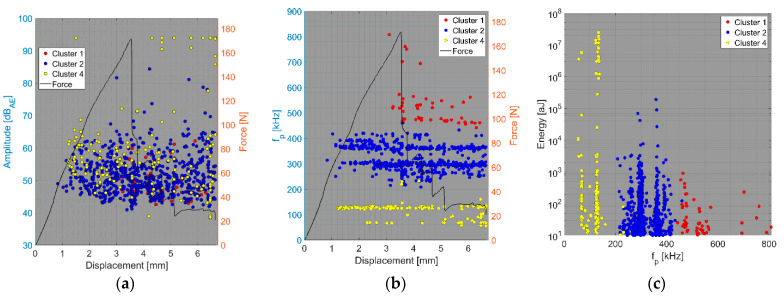
Clustering results for a CPCT representative sample using a spectral clustering algorithm (DWC 454 dataset).

**Figure 20:**
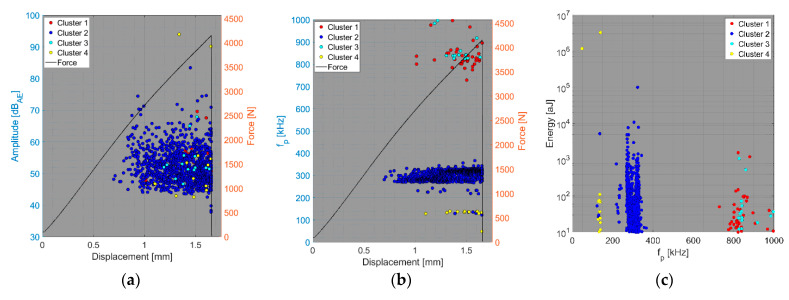
Clustering results for a CPT representative sample using a spectral clustering algorithm and secondary filtering using a partial power variable (Fujicera 1045S dataset).

**Figure 21:**
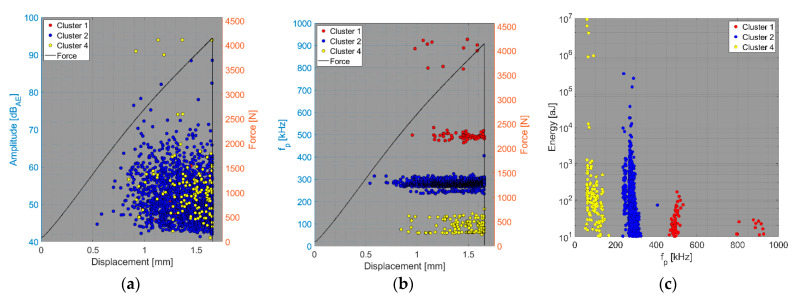
Clustering results for a CPT representative sample using a spectral clustering algorithm (DWC 454 dataset).

**Figure 22:**
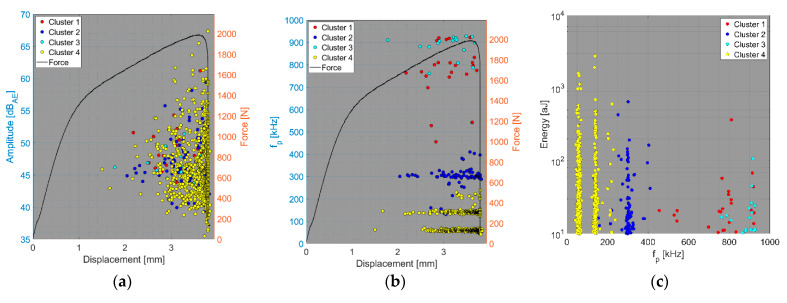
Clustering results for a CPTS representative sample using a spectral clustering algorithm and secondary filtering using a partial power variable (Fujicera 1045S dataset).

**Figure 23:**
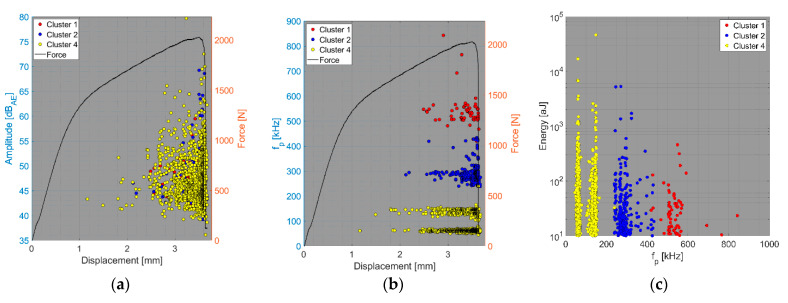
Clustering results for a CPTS representative sample using a spectral clustering algorithm (DWC 454 dataset).

**Figure 24:**
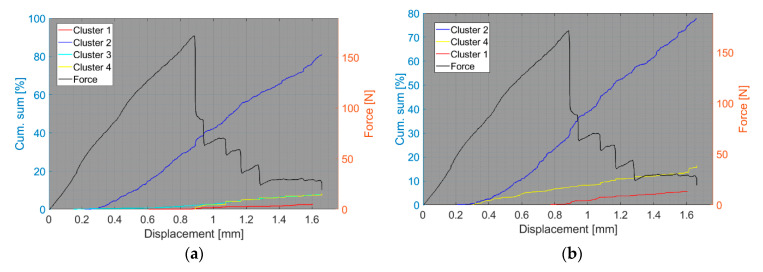
Cumulative sum of hits of individual clusters, including force versus displacement—CPCT samples: (**a**) Fujicera 1045S dataset; (**b**) DWC 454 dataset.

**Figure 25:**
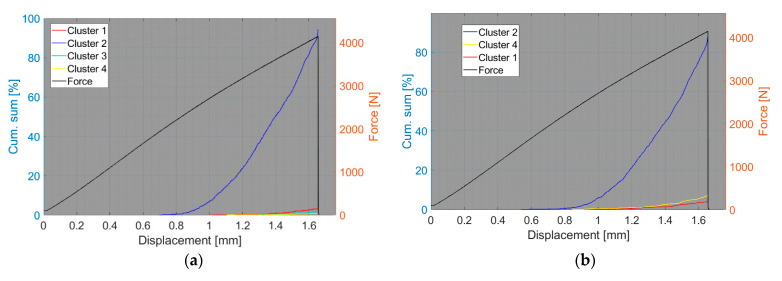
Cumulative sum of hits of individual clusters, including force versus displacement—CPT samples: (**a**) Fujicera 1045S dataset; (**b**) DWC 454 dataset.

**Figure 26:**
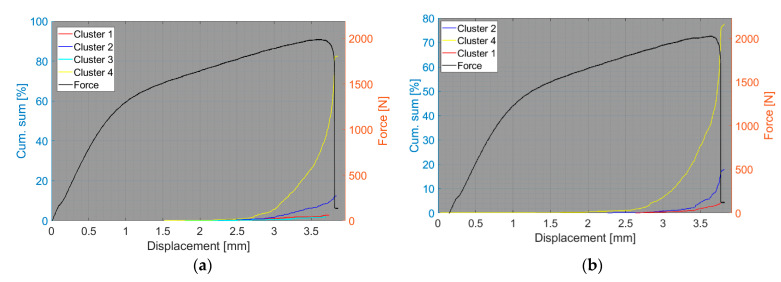
Cumulative sum of hits of individual clusters, including force versus displacement—CPTS samples: (**a**) Fujicera 1045S dataset; (**b**) DWC 454 dataset.

The results of the compact tension test of CPCT samples as well as the tensile test of CPT and CPTS samples (Figure 18, Figure 19, Figure 20, Figure 21, Figure 22, Figure 23, Figure 24, Figure 25 and Figure 26) point to the presence of five failure mechanisms, namely fibre breakage (cluster one), fibre/matrix debonding (cluster two), fibre pull-out (cluster three), matrix cracking and delamination (cluster four), which are directly related to the tested stacking sequence configuration. The CT test of the CPCT specimens revealed a somewhat different nature of the failure evolution process compared to the UCT specimens. While the AE activity of the UCT specimens was triggered by the onset of macrocrack propagation, the CPCT specimens exhibited considerable AE activity before the collapse of the structure. In addition, both datasets showed the presence of fibre/matrix debonding in the early phase, followed by matrix cracking, and fibre pull-out, especially in the case of the Fujicera 1045S dataset (see Figure 18, Figure 19 and Figure 24).

Fibre breakage (cluster one) occurred when a more significant structural collapse was present (see Figure 18b and Figure 19b). A relatively similar description of the results can be adopted in the case of the tensile test of the CPT samples (Figure 20, Figure 21 and Figure 25), where the fibre/matrix debonding mechanism and matrix cracking were even more predominant than in the case of CPCT samples. The results of the CPTS samples to a certain extent replicate those of the CPT samples, but with the difference that the predominant detected mechanism is matrix cracking (see Figure 22, Figure 23 and Figure 26).

### 4.1. Delamination and Matrix Cracking

**Figure 27:**
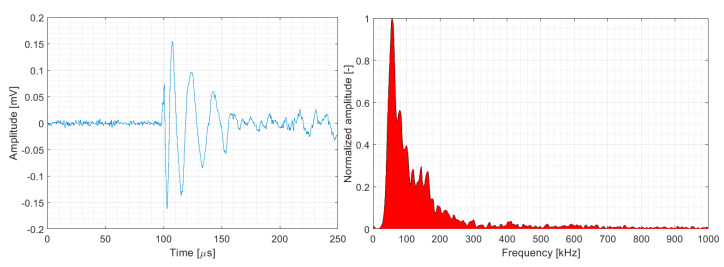
Representative AE signal of matrix cracking (DWC 454 dataset).

To the best of our knowledge, delamination is a synergy of AE signals, which are characterised by a wide spectrum of energy (up to 10^7^ aJ) and amplitude values (up to 94 dB_AE_ and occasionally more). Regarding the peak frequency, several authors have characterised this phenomenon as having a low peak frequency, often below 100 kHz [3,30,34,35]. This conclusion is partially confirmed by the results of the BRCT samples from both datasets. However, clustering results from both datasets, which are related to UCT, CPCT, CPT, and CPTS samples, clearly demonstrate that the matrix cracking/delamination phenomenon spans from 50 to approximately 200 kHz (see Figure 27).

### 4.2. Fibre/Matrix Debonding

**Figure 28:**
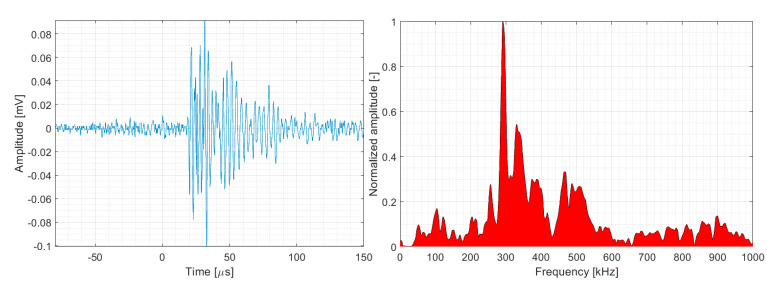
Representative AE signal of fibre/matrix debonding (DWC 454 dataset).

The reported energy and amplitude values for the fibre/matrix debonding mechanism are somewhat lower than those for delamination/matrix cracking, that is, <10^6^ aJ and <85 dB_AE_, respectively, within the maximum limiting values, while most AE hits belonging to fibre/matrix debonding have amplitude and energy values below 70 dB_AE_ and 10^5^ aJ, respectively. The published results from both datasets also confirm that both the amplitude and energy values are closely related to the loading characteristics and stacking sequence of the plies. A similar conclusion applies in the case of the peak frequency, in which the frequency interval is related to the loading characteristics and stacking sequence of the plies. The 200-400 kHz peak frequency range we found was the widest compared to those of other studies (see Figure 28) [3,33,34].

### 4.3. Fibre Failure

**Figure 29:**
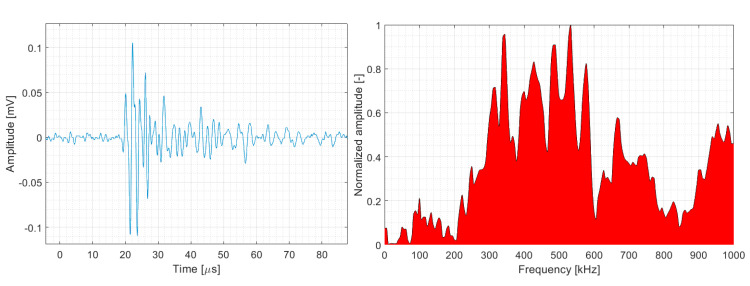
Representative AE signal of fibre failure (DWC 454 dataset).

AE hits associated with the fibre failure mechanism were characterised by energies lower than 10^4^ aJ and amplitudes up to 80 dB_AE_. As in other cases, both variables tend to be somewhat lower in the case of the Fujicera 1045S dataset. However, a major difference was found in the case of the peak frequency, where the DWC 454 dataset revealed a 400–600 kHz frequency range (see Figure 29), whereas the Fujicera 1045S dataset extended the upper bound to 1000 kHz. This observation was confirmed by the subsequent filtering of cluster three in the Fujicera 1045S dataset. By comparing the achieved results with those in Table 1, we can state that the lower limit is in accordance with the given values.

### 4.4. Fibre Pull-Out

**Figure 30:**
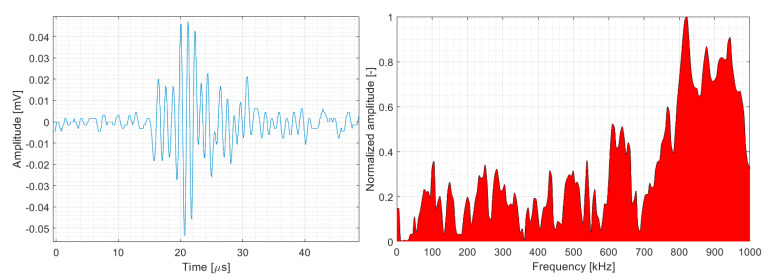
Representative AE signal of fibre pull-out (DWC 454 dataset).

Along with fibre breakage, fibre pull-out required the additional filtering of cluster three data belonging to the Fujicera 1045S dataset. This mechanism can be easily identified in the case of the DWC 454 dataset, which is part of cluster one, except for the results in the case of FCB samples. In addition, considering the filtered data from the Fujicera 1045S dataset, the AE hits belonging to fibre pull-out were mainly characterised by peak frequencies higher than 700 kHz (see Figure 30), amplitudes below 60 dB_AE_, and energies not exceeding 10^3^ aJ. The utilisation of Fujicera 1045S in similar measurements may therefore involve the estimation of the partial power of the wave transient to distinguish fibre breakage and fibre pull-out. The achieved results in terms of peak frequency are undoubtedly higher than those given in the studies listed in Table 1.

Table 5 summarises the characteristics of the reported failure mechanisms, which resulted from clustering analysis of the Fujicera 1045S and DWC 454 datasets.

## 5. Conclusions

The identification of individual failure mechanisms, including their detailed characterisation, has been the subject of a number of publications. However, many authors have only dealt with specific mechanisms from the above scale, which provided an incentive for further research in this area by our team. To obtain a more general characteristic of the individual failure modes, we used two different wideband sensors. This solution has the advantage of mutually verifying the results from both datasets.

The experimental section included the realisation of tensile or compact tension tests on bulk resin and carbon fibre bundle specimens, for which only one damage mechanism could be expected, followed by tensile/compact tension tests on uni/bidirectional ply specimens, where multiple failure mechanisms were assumed to occur.

The measured datasets were then individually subjected to cluster analysis, which involved a spectral clustering approach utilising an unsupervised k-means clustering algorithm. The feature space included besides the conventional AE signal parameters such as amplitude, energy, risetime, or frequency characteristics, non-dimensionalised AE signal power in preselected frequency intervals.

The clustered data from both datasets were then compared to each other and to the SEM analysis results. This combination proved to be very effective, especially in the case of characterising matrix cracking/delamination, or fibre/matrix debonding failure mechanisms. For a more detailed characterisation of fibre failure or fibre pull-out mechanisms in the case of the Fujicera 1045S dataset, a methodology based on secondary filtering using the non-dimensionalised AE signal power in preselected frequency intervals was proposed.

Finally, the presented method offers a robust methodology for the classification of AE signals in terms of their parameters, which benefits from the use of two different wideband sensors. The indisputable advantage of the aforementioned method is also its full applicability in industrial practice, which, however, entails the necessity to use twice the number of sensors compared to conventional technology.

## Figures and Tables

**Table 1:** Peak frequencies of AE signals (in kHz) for different failure mechanisms in CFRP composites (the results are obtained with a WD sensor).

Reference	Matrix Cracking	Delamination	Fibre/Matrix Debonding	Fibre Breakage	Fibre Pull-Out
Peak Frequency [kHz]					
[3]	80–120	120–170	170–200	~250	-
[33]	90–180	-	240–310	>300	180–240
[34]	<50	50–150	200–300	400–500	500–600
[30]	<150	150–300	-	>400	-
[35]	60–120	120–210	-	200–350	-

**Table 2:** Material properties of the test specimens.

Sample Identification	Material Parameters
Bulk resin (BRCT)	LH 385 epoxy resin with curing agent H512 E = 3.1 GPa (ex), R_m_ = 58 MPa (ex)
Unidirectional (UCT) Cross-ply (CPT, CPCT, CPTS)	carbon/epoxy prepreg CM-Preg T-C-230/600 CP004 39 nominal ply thickness = 0.25 mm, resin content = 39% E(0°) = 135 GPa (mmd), R_m_(0°) = 1900 MPa (mmd)
Carbon fibre bundle (CFB)	E = 240 GPa (mmd), R_m_ = 4 GPa (mmd)

Notes: E—Young’s modulus, Rm—Ultimate tensile strength, ex—experimentally obtained, mmd—manufacturer’s material data.

**Table 3:** Characterisation of the test specimens.

Type of Test	Name/Number of Specimens	Stacking Sequence	Thickness [mm]
Tensile test	Carbon fibre bundle (CFB)/4	-	-
Tensile test	Cross-ply (CPT)/4	(90°, 0°)_4S_	2
Tensile test	Cross-ply (CPTS)/4	(45°, −45°)_4S_	2
Compact tension	Unidirectional (UCT)/4	(90°)_4_	1
Compact tension	Cross-ply (CPCT)/4	(90°, 0°)_4S_	2
Compact tension	Bulk resin (BRCT)/4	-	5

**Table 4:** Characterisation of AE features used in this study.

Feature	Description
Amplitude (E)	Largest voltage peak of the given AE hit (in dB_AE_) (dB rel. to 1 µV before the input to the preamplifier).
Risetime (E)	Time interval (in µs) between the first threshold crossing and the reached maximum amplitude (in µs).
Duration (E)	Time interval (in µs) between the first and last threshold crossings (in µs).
Energy (E)	Integral of the squared AE signal over time ($E=\int_{D}^{\;}U{\left(t\right)}^{2}dt$, D refers to the AE hit duration) (in aJ).
*f_p_*—Peak frequency (E)	Frequency corresponding to the maximum magnitude in the frequency spectrum (in kHz).
*f_c_*—Frequency centroid (E)	Centre of mass of the frequency spectrum (in kHz).
*f_pw_*—Weighted peak frequency (C)	Square root of the product between the peak frequency and frequency centroid [37], namely $f_{pw}=\sqrt{f_{p}f_{c}}$ (in kHz).
*RA*—RA value (C) *p_fI_ ÷ p_fVI_*—Partial power (C)	Risetime/peak amplitude ratio (in µs/dB_AE_) [38]. (RA is sometimes called the rise angle). Non-dimensionalised ratio between the power in the frequency interval I÷VI and the power of the entire frequency spectrum, that is, within the ⟨50, 1100⟩ [kHz] frequency interval. Note: $f_{I}=\langle 50,225),f_{II}=\langle 225,400),f_{III}=\langle 400,575),$ $f_{IV}=\langle 575,750),$ $f_{V}=\langle 750,925),$ $f_{VI}=\langle 925,1100)$ $\left[kHz\right]$.

Note: E—extracted feature, C—calculated feature.

**Table 5:** Characterisation of reported failure mechanisms.

Failure Mechanism	A [dB_AE_]	E [aJ]	Peak Frequency [kHz]
Delamination/Matrix cracking	40÷94 (occas. 100)	<10^7^	50÷200
Fibre/Matrix debonding	40÷70 (occas. 85)	<10^5^ (occas. 10^6^)	200÷400
Fibre failure	<80	<10^4^	400÷600(1000)
Fibre pull-out	<60	<10^3^	>700

## References

[1] Šofer M., Cienciala J., Fusek M., Pavlíček P., Moravec R. (2021). Damage analysis of composite CFRP tubes using acoustic emission monitoring and pattern recognition approach. Materials.

[2] Xu J., Wang W., Han Q., Liu X. (2020). Damage pattern recognition and damage evolution analysis of unidirectional CFRP tendons under tensile loading using acoustic emission technology. Compos. Struct..

[3] Arumugam V., Sidharth A.A.P., Santulli C. (2014). Failure modes characterization of impacted carbon fibre reinforced plastics laminates under compression loading using acoustic emission. J. Compos. Mater..

[4] Dong J., Kim B., Locquet A., McKeon P., Declercq N., Citrin D.S. (2015). Nondestructive evaluation of forced delamination in glass fiber reinforced composites by terahertz and ultrasonic waves. Compos. Part B Eng..

[5] Heuer H., Schulze M., Pooch M., Gäbler S., Nocke A., Bardl G. (2015). Review on quality assurance along the CFRP value chain—Nondestructive testing of fabrics, preforms and CFRP by HF radio wave techniques. Compos. Part B Eng..

[6] Garcea S.C., Wang Y., Withers P.J. (2018). X-ray computed tomography of polymer composites. Compos. Sci. Technol..

[7] Panasiuk K., Dudzik K. (2022). Determining the stages of deformation and destruction of composite materials in a static tensile test by acoustic emission. Materials.

[8] Ferdinánd M., Várdai R., Móczó J., Pukánszky B. (2021). Deformation and failure mechanism of particulate filled and short fiber reinforced thermoplastics: Detection and analysis by acoustic emission testing. Polymers.

[9] Gholizadeh A., Mansouri H., Nikbakht A., Saghafi H., Fotouhi M. (2021). Applying acoustic emission technique for detecting various damages occurred in PCL nanomodified composite laminates. Polymers.

[10] Pacheco-Salazar O.F., Wakayama S., Can-Herrera L.A., Dzul-Cervantes M.A.A., Ríos-Soberanis C.R., Cervantes-Uc J.M. (2020). Damage evolution and fracture events sequence analysis of core-shell nanoparticle modified bone cements by acoustic emission technique. Polymers.

[11] Guo Y., Zhu S., Yuxia C., Liu D., Li D. (2019). Acoustic emission-based study to characterize the crack initiation point of wood fiber/HDPE composites. Polymers.

[12] Šofer M., Kučera P., Mazancová E., Krejčí L. (2019). Acoustic emission and fractographic analysis of seamless steel pressure cylinders with artificial flaws under hydrostatic burst testing. J. Nondestruct. Eval..

[13] Yaacoubi S., Dahmene F., Bouzenad A., el Mountassir M., Aouini M. (2018). Modal acoustic emission for composite structures health monitoring: Issues to save computing time and algorithmic implementation. Compos. Struct..

[14] Godin N., Huguet S., Gaertner R. (2005). Integration of the Kohonen’s self-organising map and k-means algorithm for the segmentation of the AE data collected during tensile tests on cross-ply composites. NDTE Int..

[15] Shateri M., Ghaib M., Svecova D., Thomson D. (2017). On acoustic emission for damage detection and failure prediction in fiber reinforced polymer rods using pattern recognition analysis. Smart Mater. Struct..

[16] Grosse C.U., Ohtsu M. (2008). Acoustic Emission Testing.

[17] Fotouhi M., Najafabadi M.A. (2014). Acoustic emission-based study to characterize the initiation of delamination in composite materials. J. Thermoplast. Compos. Mater..

[18] Sause M.G.R., Müller T., Horoschenkoff A., Horn S. (2012). Quantification of failure mechanisms in mode-I loading of fiber reinforced plastics utilizing acoustic emission analysis. Compos. Sci. Technol..

[19] Dongsheng L., Qian H., Jinping O. (2012). Fatigue damage evolution and monitoring of carbon fiber reinforced polymer bridge cable by acoustic emission technique. Int. J. Distrib. Sens. Netw..

[20] Mohammadi R., Najafabadi M.A., Saeedifar M., Yousefi J., Minak G. (2017). Correlation of acoustic emission with finite element predicted damages in open-hole tensile laminated composites. Compos. Part B Eng..

[21] Norden H.E., Zheng S., Steven R.L., Manli C.W., Hsing H.S., Quanan Z., Yen N.-C., Tung C.C., Liu H.H. (1998). The empirical mode decomposition and the hilbert spectrum for nonlinear and non-stationary time series analysis. Proc. R. Soc. Lond. Ser. A.

[22] Sause M.G.R. (2018). In-Situ Monitoring of Fiber-Reinforced Composites: Theory, Basic Concepts, Methods, and Applications.

[23] Roundi W., el Mahi A., el Gharad A., Rebiere J.-L. (2018). Acoustic emission monitoring of damage progression in Glass/Epoxy composites during static and fatigue tensile tests. Appl. Acoust..

[24] Ameur M.B., el Mahi A., Rebiere J.-L., Gimenez I., Beyaoui M., Abdennadher M., Haddar M. (2019). Investigation and identification of damage mechanisms of unidirectional carbon/flax hybrid composites using acoustic emission. Eng. Fract. Mech..

[25] Tabrizi I.E., Kefal A., Zanjani J.S.M., Akalin C., Yildiz M. (2019). Experimental and numerical investigation on fracture behavior of glass/carbon fiber hybrid composites using acoustic emission method and refined zigzag theory. Compos. Struct..

[26] Li L., Swolfs Y., Straumit I., Yan X., Lomov S.V. (2015). Cluster analysis of acoustic emission signals for 2D and 3D woven carbon fiber/epoxy composites. J. Compos. Mater..

[27] Saidane E.H., Scida D., Assarar M., Ayad R. (2017). Damage mechanisms assessment of hybrid flax-glass fibre composites using acoustic emission. Compos. Struct..

[28] Marec A., Thomas J.H., el Guerjouma R. (2008). Damage characterization of polymer-based composite materials: Multivariable analysis and wavelet transform for clustering acoustic emission data. Mech. Syst. Signal Process..

[29] Pashmforoush F., Khamedi R., Fotouhi M., Hajikhani M., Ahmadi M. (2014). Damage classification of sandwich composites using acoustic emission technique and k-means genetic algorithm. J. Nondestruct. Eval..

[30] Saeedifar M., Najafabadi M.A., Zarouchas D., Toudeshky H.H., Jalalvand M. (2018). Clustering of interlaminar and intralaminar damages in laminated composites under indentation loading using acoustic emission. Compos. Part B Eng..

[31] Oskouei A.R., Heidary H., Ahmadi M., Farajpur M. (2012). Unsupervised acoustic emission data clustering for the analysis of damage mechanisms in glass/polyester composites. Mater. Des..

[32] Saeedifar M., Zarouchas D. (2020). Damage characterization of laminated composites using acoustic emission: A review. Compos. Part B.

[33] De Groot P.J., Wijnen P.A.M., Janssen R.B.F. (1995). Real-time frequency determination of acoustic emission for different fracture mechanisms in carbon/epoxy composites. Compos. Sci. Technol..

[34] Gutkin R., Green C.J., Vangrattanachai S., Pinho S.T., Robinson P., Curtis P.T. (2011). On acoustic emission for failure investigation in CFRP: Pattern recognition and peak frequency analyses. Mech. Syst. Signal Process..

[35] Boominathan R., Arumugam V., Santulli C., Sidharth A.A.P., Sankar R.A., Sridhar B.T.N. (2014). Acoustic emission characterization of the temperature effect on falling weight impact damage in carbon/epoxy laminates. Compos. Part B Eng..

[36] Schull P.J. (2002). Nondestructive Evaluation: Theory, Techniques, and Applications.

[37] Sause M.G.R., Gribov A., Unwin A.R., Horn S., Schull P.J. (2012). Pattern recognition approach to identify natural clusters of acoustic emission signals. Pattern Recognit. Lett..

[38] Liu X., Liu Z., Li X., Cui J. (2019). Acoustic emission RA-value and granite fracture modes under dynamic and static loads. Advances in Acoustic Emission Technology.

[39] Khan R. (2019). Fiber bridging in composite laminates: A literature review. Compos. Struct..

[40] Barile C., Casavola C., Pappalettera G., Kannan V.P. (2022). Laplacian score and K-means data clustering for damage characterization of adhesively bonded CFRP composites by means of acoustic emission technique. Appl. Acoust..

[41] Ichenihi A., Li W., Gao Y., Rao Y. (2021). Feature selection and clustering of damage for pseudo-ductile unidirectional carbon/glass hybrid composite using acoustic emission. Appl. Acoust..

[42] He X., Cai D., Niyogi P. (2005). Laplacian score for feature selection. Adv. Neural Inf. Process. Syst..

[43] Li L., Lomov S.V., Yan X., Carvelli V. (2014). Cluster analysis of acoustic emission signals for 2D and 3D woven glass/epoxy composites. Compos. Struct..

[44] Alia A., Fantozzi G., Godin N., Osmani H., Reynaud P. (2019). Mechanical behaviour of jute fibre-reinforced polyester composite: Characterization of damage mechanisms using acoustic emission and microstructural observations. J. Compos. Mater..

[45] https://uk.mathworks.com/help/stats/fsulaplacian.html.

[46] Ahmed M., Seraj R., Islam S.M.S. (2020). The k-means Algorithm: A Comprehensive survey and performance evaluation. Electronics.

[47] Von Luxburg U. (2007). A Tutorial on spectral clustering. Stat. Comput..

[48] Ng A.Y., Jordan M., Weiss Y. (2001). On spectral clustering: Analysis and an algorithm. Proceedings of the Advances in Neural Information Processing Systems 14.

[49] https://www.mathworks.com/help/stats/spectralcluster.html.

[50] Chaimontree S., Atkinson K., Coenen F. (2010). Best clustering configuration metrics: Towards multiagent based clustering. Advanced Data Mining and Applications.

[51] Kaufman L., Rousseeuw P.J. (1990). Finding Groups in Data: An Introduction to Cluster Analysis.

